# Oral Yeast-Cell Microcapsule-Mediated DNA Vaccines Against *Clostridium perfringens* Induce Effective Intestinal Immunity and Modulate Gut Microbiota

**DOI:** 10.3390/vaccines12121360

**Published:** 2024-12-01

**Authors:** Lihong Du, Shaona Jia, Wenqiang Zhang, Chang Cai, Yufei Liu, Chuhan Wang, Yufei Zhu, Xiaotao Ma, Xiaojun Yang, Zehui Wei, Kun Xu

**Affiliations:** 1 Hainan Institute of Northwest A&F University, Sanya 572024, China; dlh@nwafu.edu.cn (L.D.); jiashaona@nwafu.edu.cn (S.J.); zhoulinlin@nwafu.edu.cn (W.Z.); caichang@nwafu.edu.cn (C.C.); 2019013890@nwafu.edu.cn (Y.L.); wangchuhan@nwafu.edu.cn (C.W.); 2 College of Animal Science and Technology, Northwest A&F University, Yangling 712100, China; yangxj@nwsuaf.edu.cn; 3 DAYU Bioengineering (Xi’an) Industrial Development Research Institute, Xi’an 710000, China; zhuyufei@sxdysw.cn (Y.Z.); admin@sxdysw.cn (X.M.); 4 Shanxi Dayu Biological Functions Co., Ltd., Yunchen 044000, China

**Keywords:** yeast-cell microcapsule, DNA vaccine, oral delivery, oral immunization, gut microbiota

## Abstract

**Background/Objectives:***Clostridium perfringens* is a common opportunistic pathogen that causes gastrointestinal diseases in livestock and poultry. Our preliminary research has demonstrated that administering oral yeast-cell microcapsule (YCM)-mediated DNA vaccines can effectively stimulate mucosal immunity, thereby preventing the occurrence of gastrointestinal diseases. **Methods:** In this study, the *C. perfringens* α-toxin gene was first cloned and the H126G and C-terminal (C247–370) mutations were created. The corresponding DNA vaccine cassettes driven by a CMV promoter were constructed and were cloned into a yeast shuttle vector. Recombinant yeast strains transformed with these shuttle vectors were then prepared as the YCMs for the subsequent oral immunization of mice. **Results:** Oral administration of recombinant YCMs can induce an effective immune response, and the H126G YCM performed much better than C247–370. Further evidence suggested that YCM administration may contribute to modulating the gut environment by altering gut microbiota and enhancing bacterial richness. **Conclusions:** Our study indicated that the oral administration of YCM-mediated DNA vaccines can induce effective intestinal immunity and may also alter the composition of the gut microbiota, suggesting a promising candidate vaccine strategy against *C. perfringens*-induced animal diseases.

## 1. Introduction

*Clostridium perfringens* is a significant zoonotic opportunistic pathogen that is widely present in nature and in the intestines of both humans and animals as a normal gut microbiota. It can cause muscle necrosis, necrotizing enteritis, and food poisoning in humans, as well as gastrointestinal and intestinal toxic diseases in other animals. As a result, it frequently results in substantial economic losses in animal husbandry [1,2].

The pathogenic mechanism of Clostridium perfringens primarily involves the secretion of toxins. Recent research has classified it into seven types (A, B, C, D, E, F, and G) based on the types of toxins produced [3]. All seven strains secrete α-toxin, which possesses phospholipase activity [4,5]. This toxin has been identified as the primary toxin responsible for gas gangrene and necrotizing enterocolitis, and it is one of the most lethal toxins [6]. Currently, there is no effective treatment available for severe infections caused by Clostridium perfringens, a bacterium known for its gas-producing ability. Historically, antibiotics have been used preventively and therapeutically in these situations. However, the overuse of antibiotics has become a global public health safety issue [7]. In 2020, the Ministry of Agriculture and Rural Affairs of China issued a policy document prohibiting the use of antibiotics in animal feed. As a result, it is becoming increasingly important to identify new control strategies to replace antibiotics. Vaccination has been proven to be an effective means of disease prevention. Therefore, there are numerous toxoid and subunit vaccines available for the prevention of C. perfringens which have demonstrated a certain level of protective efficacy [8].

A structural analysis of Clostridium perfringens α-toxin (CPA) indicates that it contains two active domains, an N-terminal α-helical domain with a single active site of the enzyme and a C-terminal β-sandwich domain that is crucial for cytolysis and toxicity [9]. To date, two approaches have been proposed for the development of an α-toxin vaccine. The first method involves expressing the C-terminal domain of the toxin as an immunogen [10,11], while the second method involves expressing the entire toxin that has lost its toxicity. Williamson et al. investigated the immunogenicity of CPA_1–249_ and CPA_247–370_, revealing that antibodies generated upon CPA_1–249_ immunization were capable of neutralizing phospholipase C activity in α-toxin but not its hemolytic activity. Conversely, antibodies produced upon CPA_247–370_ immunization demonstrated equal efficacy against both phospholipase C activity and hemolysis, offering complete protection [12]. Several studies have demonstrated that the C-terminal structure domain α-toxin (C_247–370_) of Clostridium perfringens, an effective antigen, can effectively induce protection against α-toxin when used in lieu of the entire CPA protein [13,14]. Furthermore, it has been reported that the replacement of histidine residues at positions 68, 126, 136, and 148 in C. perfringens α-toxin with alternative amino acids, such as glycine, can result in the inactivation of the α-toxin while preserving its immunogenicity [15].

However, there are currently no relevant research reports comparing the in vivo immune-inducing efficacy of the two target antigens used in constructing a DNA vaccine. DNA vaccination is a relatively new vaccine technology that differs from traditional protein vaccines. The DNA vaccine operates on the principle of cloning the immunogenic antigen DNA onto a plasmid and expressing it in the host cell using an efficient eukaryotic promoter. This method offers the advantages of low-cost and large-scale production. The in vivo expression of antigen genes and the translation modification of endogenous proteins result in the production of natural protein conformation, which mimics natural infection. Consequently, the antigen is presented in its native form by both MHC class I and class II complexes, thereby stimulating CD4 and CD8 T cells [16,17]. Oral administration is one of the most attractive methods for vaccine immunization. It is considered safer and easier to accept, does not cause significant stress, and can induce mucosal immunity [18]. As a result, it is favored by the majority of researchers.

In recent years, *Saccharomyces cerevisiae* has been widely utilized as a biological carrier for oral vaccines, and it is considered a safe drug delivery vehicle [19]. The oral expression of vaccine antigens by yeast can induce a strong response in mucosal-associated tissues (increased IgA secretion) and systemic (IgG) responses [20]. Moreover, relevant studies have not reported any adverse reactions with live yeast [21]. *S. cerevisiae* has a long history of safe use in human history and has been used as a feed additive [22]. Several studies have demonstrated that the use of S. cerevisiae as a feed supplement for piglets and calves can effectively regulate gut microbiota and improve intestinal homeostasis [23,24,25], while gut microorganisms have been considered to be an important factor affecting the efficacy of the vaccine in recent years [26,27,28].

The cell wall components of S. cerevisiae, such as β-glucan and mannan, are natural immune adjuvants that have immunomodulatory effects [29,30]. They can interact with pattern recognition receptors on dendritic cells (DCs), such as TLR4, TLR2, and mannose receptors, can activate DCs, and can present antigens [31,32]. These properties are essential for the use of yeast as an oral delivery vehicle [31,33], making it a potential delivery vehicle for oral vaccines. In other words, yeast-encapsulated DNA vaccines in the form of microcapsules can protect antigen genes from the erosion of digestive enzymes in the gastrointestinal tract. During our initial work, we confirmed that yeast microcapsule-mediated DNA vaccines could activate immune responses in mice [34] and fish [35]. Furthermore, we constructed a composite shRNA-MSTN type DNA vaccine YCM [36], which improved the suppression effect on endogenous MSTN in mice, providing a reference for the development of new YCM vaccines.

**Figure 1:**
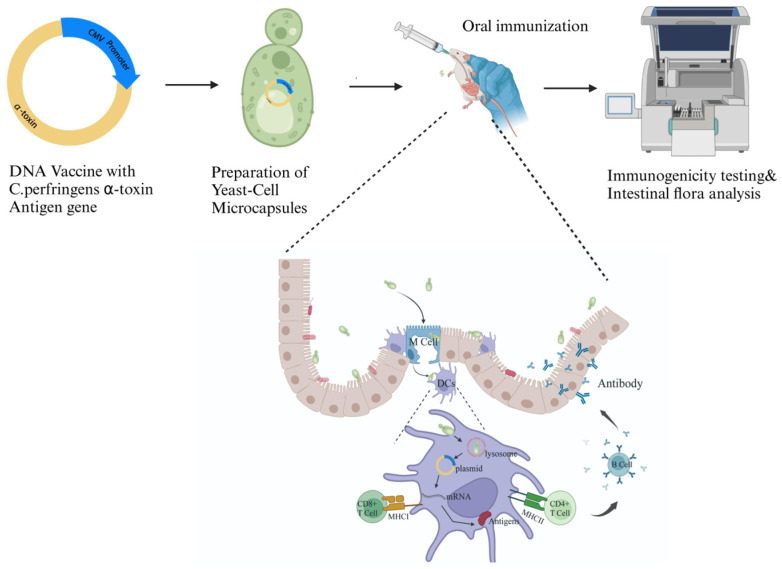
A schematic diagram of the study design. A vector carrying the gene encoding the α-toxin antigen is constructed, yeast-cell microcapsules are prepared, and their immunogenicity and their regulatory effect on the gut microbiota are evaluated.

In this study, we constructed a vector containing the antigen gene of α-toxin and transformed it into *S. cerevisiae* to produce an oral DNA vaccine, which we refer to as the yeast-cell microcapsule (YCM). Two types of YCMs were prepared in this study, the C-terminal domain of the C. perfringens α-toxin (C247–370) and the mutated whole toxin (with a histidine-to-glycine mutation at position 126). We compared the immune response and changes in intestinal microbiota induced by these different forms of antigens used as DNA vaccines to study their immunological effects (Figure 1).

## 2. Materials and Methods

### 2.1. Plasmid Construction

The alpha-toxin gene of *C. perfringens* (GenBank: AY823400.1) was synthesized by a biological company (Tsingke, Beijing, China). A fragment of the alpha-toxin with histidine 126 mutated to glycine was obtained using SOE-PCR, and a *Bam*H Ⅰ restriction site was added at the 5′ end and a *Sal* Ⅰ restriction site was added at the 3′ end. The primer sequences and PCR program are provided in the Supplementary Materials (Supplementary Table S1). The JMB84-CMV-T2A-EGFP vector (maintained in our laboratory) and the H126G fragment were digested with *Bam*H Ⅰ/*Sal* Ⅰ (Takara, Beijing, China). After gel extraction, these DNA products were ligated with T4 DNA ligase to construct the vector JMB84-CMV-CPα-H126G-T2A-EGFP. The JMB84-CMV-CPα-H126G-T2A-EGFP vector was then digested with *Sal* Ⅰ and *Xho* Ⅰ to obtain the JMB84-CMV-CPα-H126G vector. Similarly, the JMB84-CMV-T2A-EGFP vector was digested with *Sal* Ⅰ and *Xho* Ⅰ to obtain the JMB84-CMV empty vector.

The C-terminal domain fragment of the α-toxin (C247–370) was obtained through PCR, with the primer sequences and PCR program provided in Supplementary Table S2. The JMB84-CMV-CPα-C247-370-T2A-EGFP and JMB84-CMV-CPα-C247-370 vectors were constructed using the same methods as described above.

The α-toxin gene fragment was cloned into the prokaryotic expression vector pET-32a by double digestion with *Bam*H Ⅰ and *Sal* Ⅰ (TaKaRa, China) to obtain the pET-32a-CPα vector.

The details of all plasmids used in this study are listed in Table 1.

### 2.2. Testing Successful Expression of Target Vector in Eukaryotic Cells

To assess the normal expression of the constructed vector in eukaryotic cells, we transfected HEK293T cells (derived from human embryonic kidneys and transformed with large T antigens) with the vectors. HEK293T cells are a commonly used model cell line in laboratory research. The cells were cultured under standard conditions at 37 °C with 5% CO_2_. Once the cell plating density reached 85%–90%, we used Hieff TransTM Liposomal Transfection Reagent (YEASEN, Shanghai, China) to transfect the three vectors, JMB84-CMV-T2A-EGFP, JMB84-CMV-CPα-H126G-T2A-EGFP, and JMB84-CMV-CPα-C247-370-T2A-EGFP, following the manufacturer’s instructions. After 48 h of transfection, we observed the fluorescence expression of the cells using a fluorescence microscope. Subsequently, we collected the cells to determine the efficiency of transfection by flow cytometry analysis.

### 2.3. Preparation and Screning of YCM

*S. cerevisiae* JMY1 kept in our laboratory was grown aerobically in Yeast Extract Peptone Dextrose Medium (Sigma-Aldrich, St. Louis, MO, USA) at 30 °C. The eukaryotic expression vectors JMB84-CMV, JMB84-CMV-CPα-H126G, and JMB84-CMV-CPα-C247-370 were transformed into JMY1 by the lithium acetate transformation method. After cultivating for 3–4 days, single clones were picked up and cultivated in liquid culture media lacking uracil. Subsequently, the cultures were serially diluted and observed for growth patterns on uracil-deficient medium plates. To further confirm that the vector was successfully transferred to *S. cerevisiae*, we extracted the yeast plasmid using the yeast extraction kit (Solarbio, Beijing, China) and transferred it to *E. coli* JM109. After culturing in LB medium containing ampicillin, the plasmid was extracted from JM109 using the plasmid extraction kit (Omega Bio-tek, Norcross, GA, USA) and identified by double restriction enzyme digestion. The YCM carrying the expressed vectors were named JMB84, H126G, and C247-370, respectively.

### 2.4. Prokaryotic Expression and Purification of Target Antigen Protein

The constructed pET-32a-CPα vector was transformed into *E. coli* BL21(DE3) by chemical transformation. After the cells reached an optical density of 0.8 at OD600, 100 mM IPTG was added to a final concentration of 0.5 mM, and then at 18 °C, it was shaken for 12 h to induce antigen protein expression. Next, bacterial cells were collected by centrifuging at 12,000× *g* at 4 °C for 5 min. The cell pellets were then re-suspended in phosphate-buffered saline buffer (PBS) and the suspension was subjected to sonication. Afterwards, the supernatant was collected by centrifugation at 12,000× *g* at 4 °C for 10 min. We used SDS-PAGE to confirm the successful expression of the antigen protein. The collected supernatant was added to 5× SDS loading buffer and boiled for 10 min, then subjected to SDS-PAGE in a 12% concentration. Gels were run at 150 V for 1 h in Tris-acetate buffer. Staining with Coomassie Brilliant Blue on the gel was performed to observe the expression levels. After confirming that the antigen protein was successfully expressed, we used the His TALON^TM^ Gravity Column Purification Kit (Clontech, Mountain View, CA, USA) to purify it according to the instructions. After that, we used a Millipore ultrafiltration tube to concentrate the protein purified in the previous step and dissolved it in PBS to achieve the purpose of replacing the antigen protein buffer. Finally, the BCA Protein Assay Kit (Solarbio, China) was used to determine protein concentration according to the instructions, and the protein was stored at −80 °C. The purified protein was used for the encapsulation of ELISA plates in subsequent experiments to detect specific antibodies in the sample.

### 2.5. Oral Immunization

Five-week-old female Kunming mice, weighing approximately 35 g each, purchased from Chengdu Dashuo Experimental Animal Company (Chengdu, China), were randomly divided into four groups (n = 21 per group) as follows: the PBS control group, JMB84 group (JMYI contains JMB84-CMV vector), H126G group (JMY1 contains JMB84-CMV-H126G vector), and C247–370 group (JMY1 contains JMB84-CMV-CPα-C247-370 vector). All groups were provided with a normal diet and water. The experiment was conducted after the mice adapted to the feeding environment. The recombinant *S. cerevisiae* was cultured and centrifuged, washed with PBS, and re-suspended in PBS at a concentration of 5 × 10^10^ cfu/mL. Two different vaccine groups and the empty vector group mice received 200 μL of the corresponding recombinant yeast, and the PBS control group mice received the same amount of PBS.

The immune and sampling procedures for experimental mice were conducted in accordance with preceding instructions [37]. In brief, all mice were immunized orally, and the immune protocol was repeated on three consecutive days, once every seven days, for a total of six immunizations.

### 2.6. Detection of IgG, sIgA, and Cytokines by ELISA

On day 0, 7, 14, 21, 28, 35, and 42 after immunization, serum and intestinal mucus were collected from three mice selected randomly in each group. In addition, mice on day 0 and day 42 also needed to have fecal samples collected from them. Fecal samples were quick-frozen with liquid nitrogen and stored at −80 °C. Subsequently, serum α-toxin-specific IgG and intestinal mucus α-toxin-specific secretory IgA were detected by ELISA. In brief, first it was coated with 6 μg/mL of purified α-toxin antigen protein in a polystyrene microtiter plate at 4 °C overnight. Next, it was washed three times with washing solution and blocked with blocking solution for 1 h. Then, the collected serum (diluted at 1:100) and intestinal mucus (diluted at 1:100) were used as the primary antibody, and HRP-conjected goat anti-mouse IgA or IgG (CWBIO, Taizhou, China) was used as the secondary antibody, followed by color development using TMB (Solarbio, China) as the substrate. In addition, absorbance was measured at 450 nm (the reference wavelength is 630 nm).

### 2.7. RNA Extraction and qRT-PCR

We collected the colon tissues of three mice in each treatment group on the last day and used an RNA extraction kit (CWBIO, China) to extract total RNA. Total RNA (1 μg) was reversely transcribed to cDNA using the PerfectStart^®^ Uni RT&qPCR Kit (TransGen Biotech, Beijing, China), following the manufacturer’s protocol. The qRT-PCR analysis was carried out using PerfectStart^®^ Green qPCR SuperMix on the LightCycler Real-Time PCR system of Roche. Relative expressions of genes were calculated using the 2^−ΔΔCT^method. The β-actin protein was used for gene expression normalization. The primers are listed in Supplementary Materials (Supplementary Table S3).

### 2.8. 16S rRNA Sequencing of Gut Microbiota

The mice fecal samples collected above were handed over to the Tsingke Company (Beijing, China) for 16s rRNA sequencing. The fecal samples of three mice in each treatment group were analyzed using mixed detection. Microbial diversity was based on the Illumina Novaseq sequencing platform using the paired-end method to construct small fragment libraries for sequencing.

### 2.9. Statistical Analysis

The data were presented as mean ± standard deviation (SD). All the statistical analyses were performed using GraphPad Prism V5.01 (GraphPad, Boston, MA, USA). Normality and lognormality tests were employed for assessing normality, followed by a one-way ANOVA for multiple comparisons. A Tukey post hoc test was then conducted for comprehensive pairwise comparison. *p*-values less than 0.05 and less than 0.01 were considered statistically significant and extremely significant, respectively.

## 3. Results

### 3.1. Construction and Expression of Eukaryotic Vectors

**Figure 2:**
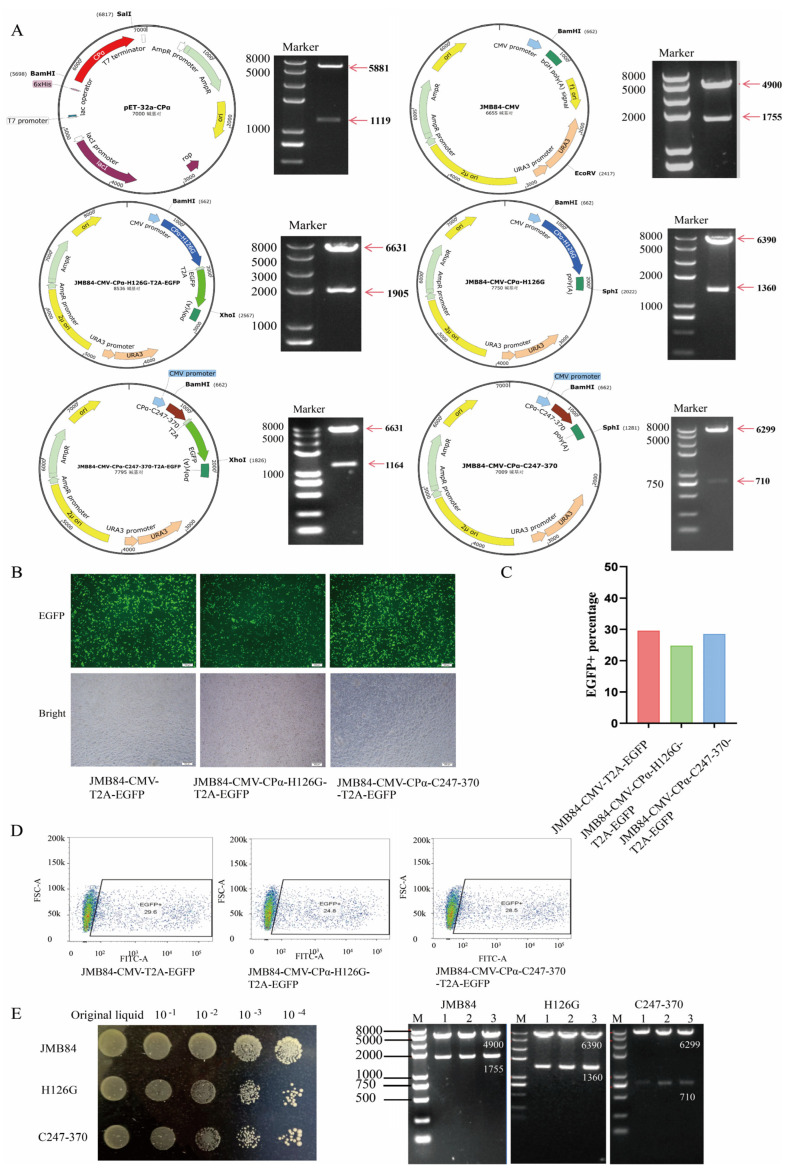
Vector expression and YCM selection identification. (**A**) Experimental vector required pET32−a−CPα, JMB84−CMV, JMB84−CMV−CPα−H126G−T2A−EGFP, JMB84−CMV−CPα−H126G, JMB84−CMV−CPα−C247−370−T2A−EGFP, and JMB84−CMV−CPα−C247−370 enzyme digestion identification; (**B**) observation of the expression level of the eukaryotic expression vector in HEK293T cells under a microscope; (**C**) percentage of EGFP fluorescent cells analyzed by flow cytometry; (**D**) proportion of EGFP-positive cells; (**E**) identification and screening of positive yeast strains.

The schematic diagrams of all vectors constructed in this study are shown in Figure 2A, and their structures were confirmed by restriction enzyme digestion analysis and DNA sequencing.

The HEK293T cells were transfected with JMB84-CMV-CPα-H126G-T2A-EGFP and JMB84-CMV-CPα-C247-370-T2A-EGFP vectors, and the JMB84-CMV-T2A-EGFP vector was used as a control. Fluorescence observation initially confirmed the successful expression of the target gene (Figure 2B). Furthermore, flow cytometry detection was performed to determine the expression efficiency of vectors constructed with different genes of interest (Figure 2C). By combining fluorescence imaging and flow cytometry data, it can be concluded that there is no significant difference in expression levels between JMB84-CMV-CPα-H126G-T2A-EGFP and JMB84-CMV-CPα-C247-370-T2A-EGFP compared to the empty vector control JMB84-CMV-T2A-EGFP (Figure 2D).

### 3.2. YCM Screening and Identification

The expression vectors JMB84-CMV, JMB84-CMV-CPα-H126G, and JMB84-CMV-CPα-C247-370 were transformed into yeast strain JMY1 using the PEG-LiAc transformation method. Positive colonies were selected on uracil-deficient SD medium and cultured to an OD600 ≈ 1.0. The cultures were then serially diluted 10-fold to create four gradients, from which 10 μL was plated from each gradient (Figure 2E). Three single colonies were picked and shaken until turbid, the yeast plasmids were extracted, and enzyme digestion identification was performed. Strain JMB84 yielded bands of 1755 bp and 4900 bp when digested with *Bam*H Ⅰ and *Eco*R Ⅴ, strain H126G yielded bands of 1360 bp and 6399 bp when digested with *Bam*H Ⅰ and *Sph* Ⅰ, and strain C247–370 yielded bands of 710 bp and 6299 bp when digested with *Bam*H Ⅰ and *Kpn* Ⅰ (Figure 2E).

### 3.3. Detection of Cpα Protein Prokaryotic Induction Expression

**Figure 3:**
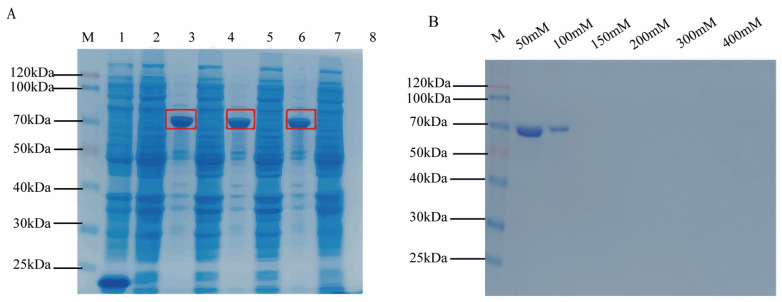
Detection and purification of α-toxin antigen proteins. (**A**) Following induction with IPTG, the antigenic protein is detected via SDS-PAGE, revealing the target protein to be 62.1 kDa in size(Mark the red squares in the diagram). Lane M, Blue Plus II protein marker (TransGen Biotech, China); lane 1, 2, pET-32a (empty vector), after and before inducible expression; lane 3, 4, pET-32a-CPα, after and before inducible expression; lanes 5–8 are experimental repetitions. (**B**) Different lanes in the SDS-PAGE gel image of alpha antigen purification represent different imidazole elution concentrations.

The prokaryotic expression of the α-toxin gene produced the corresponding protein with a size of approximately 62.1 kDa. The SDS-PAGE electrophoresis result of the prokaryotic expression protein was shown in Figure 3A. The purification results of SDS-PAGE are shown in Figure 3B, indicating that when eluted with 50 mM imidazole, most of the CPα protein can be eluted; when eluted with 100 mM imidazole, all the CPα protein in the column has been eluted clean, and no protein was detected in the eluate of subsequent concentrations of imidazole. Finally, the CPα protein in the eluate was concentrated using a Millipore ultrafiltration tube, dissolved in PBS to remove the imidazole-containing eluate. The CPα protein was dissolved in PBS buffer for subsequent use in ELISA experiments.

### 3.4. Specific Immune Response Induced by Oral Yeast Microcapsule-Mediated DNA Vaccine in Mice

We collected sera and small intestine samples of mice in accordance with the sampling procedure outlined in Figure S1. The immunogenicity of recombinant *S. cerevisiae* in mice after oral immunization was evaluated by ELISA, which mainly detected serum IgG and mucosal sIgA.

**Figure 4:**
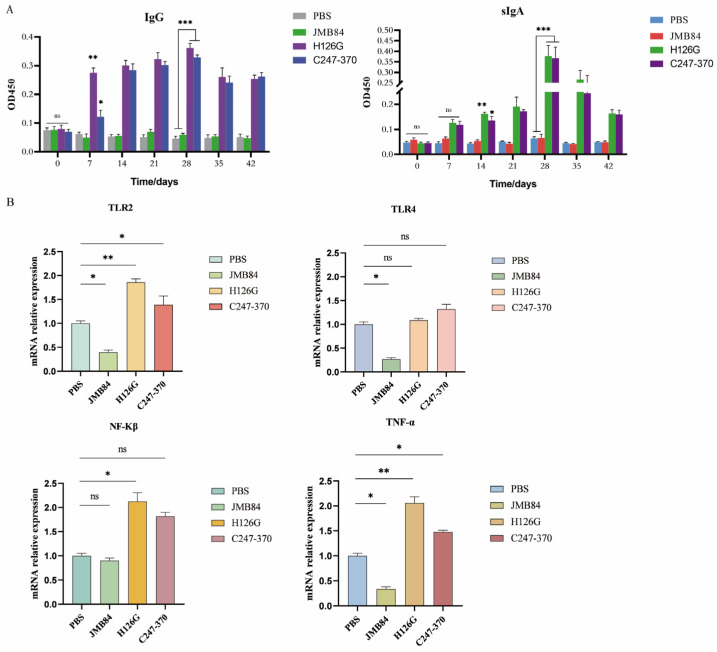
The detection of antibodies and colon-related factors. (**A**) The detection of serum-specific IgG and intestinal mucus-specific sIgA in mice. The serum-specific antibody IgG and the intestinal mucus-specific antibody sIgA were detected by ELISA at 0, 7, 14, 21, 28, 35, and 42 days after immunization in PBS, JMB84, H126G, and C247–370 groups, respectively. Results are presented as mean ± SD. ^ns^ *p* > 0.05, *** *p* < 0.0005 compared with PBS and JMB84 groups. (**B**) qRT-PCR analysis of mice colon tissues. The H126G and C247–370 groups increased the mRNA levels of TLR2, TLR4, NF-kβ, and TNF-α in colon tissues, and the empty vector yeast group (the JMB84 group) downregulated mRNA levels. Dates are expressed as the mean ± SD (n = 3). ^ns^ *p* > 0.05, * *p* < 0.05, ** *p* < 0.001 compared with the PBS group.

There was no significant difference in sIgA and IgG antibody levels among the groups before immunization (*p* > 0.05). With the increase in the numbers of immunizations, the levels of serum α-toxin-specific IgG antibody and the intestinal mucus α-toxin-specific secretory IgA antibody (Figure 4A) of the two vaccine groups were significantly higher than those in the empty vector group and the PBS control group (*p* < 0.0005). The H126G group is always higher than the C247–370 group. During the entire experiment, no adverse reactions and deaths of the mice were observed. Interestingly, the levels of IgG and IgA reached their peak simultaneously on the 28th day of the entire immune period.

### 3.5. Oral YCM DNA Vaccines Activate Mouse Immune Responses via the TLR2 Signaling Pathway

Research suggests that intestinal dendritic cells (DCs) mainly recognize Saccharomyces cerevisiae through Toll-like receptors TLR2 and TLR4. The expression levels of TLR2 and TLR4 can somewhat reflect the recognition ability of DCs towards the yeast. This study detected the mRNA expression levels of TLR2 and TLR4 in colonic tissue using qPCR. As is shown in Figure 4B, compared with the PBS control group, the mRNA levels of TNF-α, TLR2, and TLR4 in the colon tissue of mice fed with an empty vector-carrying YCM were decreased; it is suggested that yeast microcapsules carrying empty carriers may reduce the expression of pro-inflammatory cytokine TNF-α by inhibiting the expression of TLR4 and TLR2, which indicated that the YCM downregulates the inflammation of intestinal tissue, which also explains the anti-inflammatory effect of *S. cerevisiae* as a probiotic (*p* < 0.05). Studies have speculated that yeast exerts a powerful anti-inflammatory effect, perhaps because yeast is fermented by the microbiota in the caecum and colon to produce biologically active SCFAs [38]. However, the levels of TLR2, NF-kβ, and TNF-α mRNA in both vaccine groups increased (*p* < 0.05), indicating that the carried antigen genes activated NF-kβ by upregulating TLR2 genes and secreting TNF-α, resulting in an immune response. Because the TLR is a typical pattern recognition receptor, the ability to distinguish pathogen types needs to be transformed into appropriate innate and adaptive responses by selectively activating NF-κβ [39]. The dendritic cells, as the main antigen-presenting cell, can be used to sense tissue infection in the process of maturing into APC, and it is also necessary to send a signal through the NF-κβ pathway [40]. Interestingly, the levels of TLR4 mRNA in the two vaccine groups were not significantly different from those in the control group (*p* > 0.05).

### 3.6. Gut Microbiota Composition Changes After Oral Immunization

**Figure 5:**
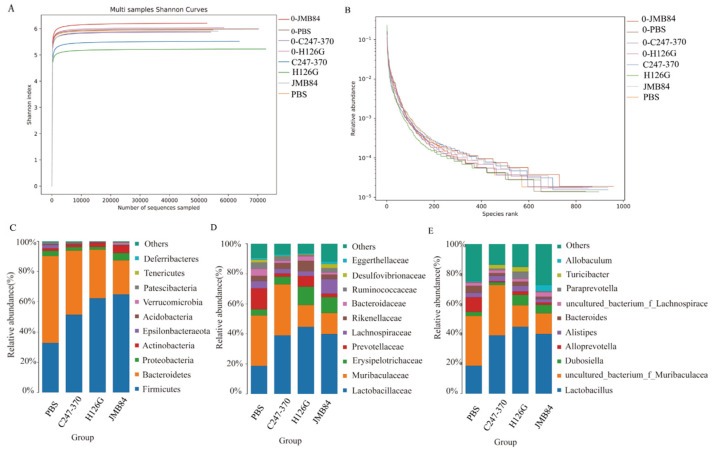
Changes in the gut microbiota of mice after oral immunization. (**A**) Analyzed alpha diversity through Shannon index. (**B**) Rank abundance curve to explain abundance and uniformity of species contained in samples. Relative abundance of top 10 abundant bacteria at phylum (**C**), family (**D**), and genus (**E**) levels.

The Shannon–Wiener curve, constructed by utilizing the Shannon index, reflects the microbial diversity in the sample, which is constructed by creating the index of microbial diversity at different sequencing depths for each sample. This curve thus represents the microbial diversity in each sample across varying sequencing quantities. As the curve tends towards flatness, it indicates that the sequencing data volume is ample enough to reflect the vast majority of microbial species information within the sample [41]. The rank abundance curve is employed to elucidate the richness and evenness of species contained within a sample. The richness of species is reflected by the length of the curve along the horizontal axis; the wider the curve, the more diverse the composition of species. The uniformity of species composition is indicated by the shape of the curve; the flatter the curve, the higher the degree of uniformity in species composition [42]. The results indicate that the sample sequencing data is reasonable and that most of the bacterial diversity has been captured in all samples. Furthermore, the Shannon indices indicated that the microbial diversity in each group decreased following the oral administration of recombinant brewing yeast in comparison to the pre-immune (“0”) and PBS groups. A comparison between the empty vector recombinant yeast group (JMB84) and the two experimental groups (H126G and C247–370) revealed lower Shannon indices in the latter, suggesting a more distinct composition of fecal microbiota in these groups. (Figure 5A,B).

In order to further understand the specific changes in the microbial community, we analyzed the differences between each group of samples at the phylum, family, and genus levels.

At the phylum level (Figure 5C), yeast modulated gut microbiota composition by increasing the ratio of thick-walled fungi/toxigenic bacteria, which is consistent with previous studies [20]. At the family level, the proportion of Lactobacillaceae was predominant in the three yeast-fed groups, but the proportion of Muribaculaceae was reduced (Figure 5D). At the phylum level, the bacteria with the highest proportions were mainly Lactobacillus, uncultured_ bacterium _f_ Muribaculaceae, Bacteroides genus, and Alloprevotella (Figure 5E). A detailed analysis at the genus level indicates that the oral administration of brewer’s yeast significantly upregulated or downregulated the relative abundance of certain bacteria. Specifically, the three groups fed recombinant brewer’s yeast showed a significant increase in the relative abundance of Lactobacillus and Dubosiella, while the abundance of uncultured Muribaculaceae and Helicobacter was decreased. More importantly, the H126G group with higher antibody titer showed higher abundance of alloprevotella compared with the C247-370 group. These results indicate that brewer’s yeast does not disrupt the balance of intestinal microbiota but rather can enhance the proportion of beneficial bacteria.

## 4. Discussion

*C. perfringens* is a common pathogenic bacterium in livestock and poultry breeding, and it causes a massive number of deaths of animals every year; thus, it is regarded as a potential threat to the development of animal husbandry because the changes in dietary composition, excessive use of antibiotics, or infection with other pathogens cause an imbalance of the intestinal flora. The intestinal environment helps the rapid reproduction of *C. perfringens*, reaching a pathogenic level, allowing it to enter the intestinal mucosa to induce diseases [4].

Vaccination is an effective means to prevent diseases caused by *C. perfringens* [43]. The use of vaccines to protect cattle, sheep, and chickens from the infection of *C. perfringens* is of great significance to the stable and healthy development of the breeding industry.

DNA vaccines have been confirmed in a variety of animal model experiments to show good preventive and therapeutic effects against viruses and various cancers [44,45]. However, insufficient immunogenicity that limits the practical application of DNA vaccines is still a huge challenge. At present, in order to improve the immunogenicity of DNA vaccines, different methods have been tried, like vector design, antigen codon optimization, and searching for more suitable delivery methods and better immune adjuvants [46].

How to better achieve the oral delivery of antigen genes through the harsh gastrointestinal environment is the key to DNA vaccines [46]. Studies have found that *S. cerevisiae* can withstand a simulated human digestive environment, which is very important for the oral delivery of vaccines, like a microcapsule, encapsulating an antigen gene expression cassette within yeast cells, then effectively delivering biomolecules to intestinal DCs. Recognizing and swallowing *S. cerevisiae* through Dectin-1 and Toll-like receptors on DCs induces the maturation of DCs [47]. *S. cerevisiae* is by far the most understood eukaryote at the molecular level and is widely used to express foreign genes and produce therapeutic proteins [48]. Its growth is simple, safe, and non-toxic, and it may be further developed into an edible vaccine [49]. Therefore, the strategy of oral immunization has great potential using a recombinant *S. cerevisiae* vaccine.

In this study, the *S. cerevisiae* expression vector, JMB84-CMV, constructed in our laboratory was used to express the antigen gene of the *C. perfringens* α-toxin. All eukaryotic expression vectors constructed in this study used the CMV promoter, which is a viral promoter commonly used in mammalian cells. Previous research conducted in our laboratory has demonstrated the strong functionality of CMV promoters derived from human cytomegalovirus in mammals. These promoters exhibit limited functionality in yeast but are capable of expressing genes in animal intestines [34]. In this study, Kunming mice were used as an animal model to evaluate the immunogenicity of two different forms of DNA vaccines with α-toxin antigen genes based on *S. cerevisiae* from the aspects of inducing mucosal- and systemic-specific antibody responses to α-toxin.

Our preliminary data indicate that mice administered orally exhibit specific mucosal antibodies, sIgA, detectable in intestinal tissue fluid, as well as IgG detectable in serum. In contrast, the 126-point mutation form as an α-toxin antigen gene appears to have a more potent effect. Therefore, the site-directed mutation target gene may be a better choice as a vaccine antigen.

In this study, besides assessing the immune response of an oral YCM-mediated DNA vaccine, we also discussed the effects of intestinal flora on an oral recombinant *S. cerevisiae* DNA vaccine. Gut microbiota has been shown to have an impact on the immune response in humans, mice, and pigs [27,50,51,52]. At the phylum level, consistent with previous studies [23], *S. cerevisiae* changed the composition of the gut microbiota by increasing the ratio of Firmicutes/Bacteroides. Specifically, we found that there were some differences in the intestinal flora between the two vaccine groups. Compared with the C247–370 group, the H126G group had higher counts of Prevotellaceae and Muribaculaceae and could induce higher immunity. This was consistent with the viewpoint reported in this article that the abundance of the genus Prevotella and the family Muribaculaceae was associated with a strong vaccine response [27].

We found that the intestinal microbiota of the C247–370 group mice with a lower antibody response was more diversified. Praharaj et al. found that the microflora of those who neutralized non-responding antibodies was more complex and diverse than those of responders [53]. Ting’s et al. study also found that the diversity of intestinal flora was negatively correlated with immune response during vaccination [51].

The data provided in this article illustrated the impact of an oral yeast microcapsule-mediated DNA vaccine on the intestinal flora for the first time and further supported that a higher antibody response was related to a more complex diversity of intestinal flora. This immunization method could be used to prevent related diseases induced by intestinal pathogens. The exploration of the intestinal flora–vaccine interaction provided new ideas for improving the efficiency of probiotic DNA vaccines.

However, there are still some limitations in our research. Although we used two different forms of DNA vaccines against the same toxin, the changes in the intestinal flora between the two vaccine groups might also be caused by differences in antigen genes or variations in polypeptide expression levels, and the sample size was too small to make a comparison within the group. Future research should include a quantitative analysis of peptide expression levels to determine their impact on changes in intestinal microbiota and vaccine efficacy. In addition, larger sample sizes and further comparative studies are needed to elucidate these relationships. However, Zhong et al. studied the effect of GLP-2 expressing *S. cerevisiae* on the intestinal microbes of weaned piglets and found that the changes in the fecal microbes of weaned piglets were caused by *S. cerevisiae* rather than GLP-2 expressed by *S. cerevisiae*. In short, the importance of the gut microbiota to the immune response of the vaccine is being studied in depth, but there is no clear conclusion in this field, so more studies are needed to explain this complex relationship.

## 5. Conclusions

In summary, our research suggests that the engineered α-toxin with point mutations may possess a stronger immunogenic potential compared to the C-terminal domain as an antigen. The oral administration of YCM can effectively activate the immune response in the body and modulate the gut microbiota, increasing the proportion of beneficial bacteria and reducing harmful ones. Therefore, it can provide better immune protection to the animal body. Furthermore, the level of antibodies produced by the body is indeed influenced by the gut microbiota. Additionally, recombinant *S. cerevisiae* has no safety risks and can be easily produced on a large scale, making it highly promising for development and application. Specifically, it can be utilized as an oral biological agent for the prevention of intestinal diseases induced by *C. perfringens* in livestock and poultry breeding, particularly in light of the current era of antibiotic bans.

## Figures and Tables

**Table 1:** Details of the plasmids used in this study.

Plasmids	Relevant Characteristic	Resistance Marker (Reference)
JMB84-CMV-T2A-EGFP	An empty vector carrying EGFP composed of CMV strong promoter and T2A cleavable peptide	AmpR, URA3; (In our lab)
JMB84-CMV-CPα- H126G-T2A-EGFP	Expressed CPα-H126G with EGFP Used to verify expression efficiency	AmpR, URA3; (In this work)
JMB84-CMV-CPα- C247-370-T2A-EGFP	Expressed CPα C-terminal domain With EGFP, used to verify expression Efficiency	AmpR,URA3; (In this work)
JMB84-CMV	Empty vector without EGFP	AmpR,URA3; (In this work)
JMB84-CMV-CPα-H126G	Expressed CPα-H126G without EGFP, used to construct recombinant yeast	AmpR,URA3; (In this work)
JMB84-CMV-CPα- C247-370	Expressed CPα C-terminal domain without EGFP, used to construct recombinant yeast	AmpR,URA3; (In this work)
pET-32a	Prokaryotic expression empty vector	AmpR; (In our lab)
pET-32a-CPα	Prokaryotic expression vector for producing α-toxin protein	AmpR; (In this work)

## Data Availability

All the raw and analysed data generated during the study are available from the corresponding authors on reasonable request.

## Supplementary Materials

The following supporting information can be downloaded at: https://www.mdpi.com/article/10.3390/vaccines12121360/s1, Figure S1: Mouse oral immunization and sampling procedures; Table S1: Primers used to obtain the α-toxin H126G fragment. Table S2: Primers required to obtain the α-toxin C-terminal domain (C247-370) fragment. Table S3:Quantitative PCR primer sequences.
