# Supplementary material for: Oral Yeast-Cell Microcapsule-Mediated DNA Vaccines Against Clostridium perfringens Induce Effective Intestinal Immunity and Modulate Gut Microbiota
Source: Vaccines (Basel). 2024 Dec 1;12(12):1360. doi: 10.3390/vaccines12121360 (PMC11680129; doi:10.3390/vaccines12121360)
Supplement: Supplementary file 1 [file vaccines-12-01360-s001.zip › vaccines-3264440-supplementary.pdf]

Supplementary materials

Table S1. Primers used to obtain the α-toxin H126G fragment (two restriction enzyme sites, *BamH*I and *Sal*I were shown in capital italics).

Primer Name	Primer Sequence
Primer a	cGGATCCatgtgggacggaaagatcgacggc
Primer b	cggcgaggccatgggctacttcggcgac
Primer c	gtcgccgaagtagcccatggcctcgccg
Primer d	gcGTCGACcttgatgttgtaggtgctg

First used primers a and c, primers b and d to obtain fragments M and N by PCR, respectively. Then, used M and N fragments as templates, primer a and d to perform PCR to obtain the final α-toxin H126G fragment. The PCR conditions were 5 minutes at 94 C for initial denaturation, 30 seconds at 94°C for denaturation, 30 seconds at 65°C for annealing, 1 minutes at 72°C for extension, repeat for total 25 cycles, 72°C for 5 minutes for final extension.

Table S2. Primers required to obtain the α-toxin C-terminal domain (C247-370) fragment (two restriction enzyme sites, *Bam*HI and *Sal*I were shown in capital italics).

Primer Name	Primer Sequence
Primer 1	ccgGGATCCtacgccgccaaggtgaccctg
Primer 2	gcGTCGACgttttctggcttgtaggcgtc

The PCR program was as above.

Table S3. Quantitative PCR primer sequences.

Primer Name	Primer Sequence
TLR2, forward primer	ACCCGCCCTTTAAGCTGTGT
TLR2,reverse primer	TCGTACTTGCACCACTCGCT
TLR4, forward primer	TCTGGGGAGGCACATCTTCT
TLR4,reverse primer	AGGTCCAAGTTGCCGTTTCT
NF-kβ, forward primer	GAGGTCTCTGGGGGTACCAT
NF-kβ,reverse primer	TTGCGGAAGGATGTCTCCAC
TNF-α, forward primer	CCAGCCGATGGGTTGTACCT
TNF-α,reverse primer	TGACGGCAGAGAGGAGGTTG
β-actin, forward primer	ATGCTCTCCCTCACGCCATC
β-actin,reverse primer	GAGGAAGAGGATGCGGCAGT

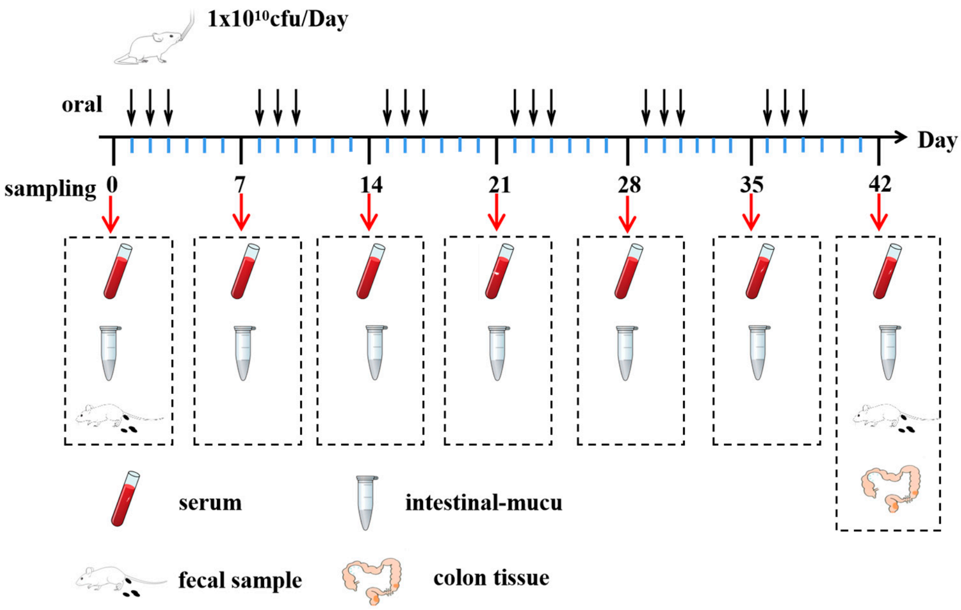

Figure S1. Mouse oral immunization and sampling procedures. Experimental mice were gavaged once a week for three consecutive days, as indicated by the black arrow; samples were collected once a week, as indicated by the red arrow.
